# Supplementary material for: Antagonization of OX1 Receptor Potentiates CB2 Receptor Function in Microglia from APPSw/Ind Mice Model
Source: Int J Mol Sci. 2022 Oct 24;23(21):12801. doi: 10.3390/ijms232112801 (PMC9656664; doi:10.3390/ijms232112801)
Supplement: Supplementary file 1 [file ijms-23-12801-s001.zip › ijms-1888592-supplementary.pdf]

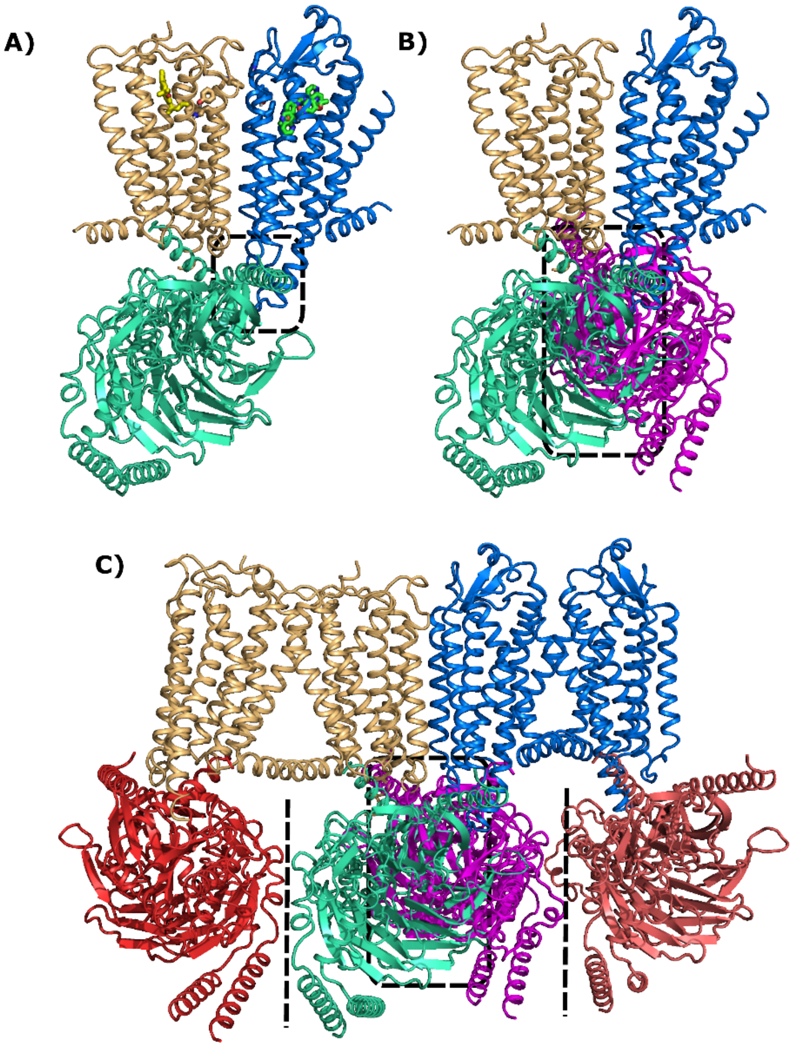

Supplementary Figure S1. How many proteins can fit a CB$_2$R-OX$_1$R heteromer? Computational model of the heteromeric TM4/5 CB$_2$R/JWH133.OX$_1$R/SB334867 complex viewed from the membrane. A) CB$_2$R receptor is shown in gold, OX$_1$R receptor in blue and a superposed G-protein in green. The heteromer would fit one G protein with only minor changes in the loop connecting TMs 3 and 4, and the end of TM6, or a small rotation of the G protein. B) An additional G-protein (in purple) would clash sterically with the first G-protein if bound simultaneously. C) In a tetramer it is possible to bind additional proteins. As an example the panel displays homodimers modeled using symmetric TM1 interfaces taken from the κ-opioid receptor (PDB id 4DJH) that could bind two additional G-proteins.
